# Androgen-Regulated Cardiac Metabolism in Aging Men

**DOI:** 10.3389/fendo.2020.00316

**Published:** 2020-05-15

**Authors:** Genaro Barrientos, Paola Llanos, Carla Basualto-Alarcón, Manuel Estrada

**Affiliations:** ^1^Programa de Fisiología y Biofísica, Facultad de Medicina, Instituto de Ciencias Biomédicas, Universidad de Chile, Santiago, Chile; ^2^Centro de Estudios en Ejercicio, Metabolismo y Cáncer (CEMC), Universidad de Chile, Santiago, Chile; ^3^Facultad de Odontología, Instituto de Investigación en Ciencias Odontológicas (ICOD), Universidad de Chile, Santiago, Chile; ^4^Departamento de Ciencias de la Salud, Universidad de Aysén, Coyhaique, Chile; ^5^Departamento de Anatomía y Medicina Legal, Facultad de Medicina, Universidad de Chile, Santiago, Chile

**Keywords:** testosterone, cardiac diseases, aging men, cardiac metabolism, glycolysis, AMPK, PGC1α, sirtuins

## Abstract

The prevalence of cardiovascular mortality is higher in men than in age-matched premenopausal women. Gender differences are linked to circulating sex-related steroid hormone levels and their cardio-specific actions, which are critical factors involved in the prevalence and features of age-associated cardiovascular disease. In women, estrogens have been described as cardioprotective agents, while in men, testosterone is the main sex steroid hormone. The effects of testosterone as a metabolic regulator and cardioprotective agent in aging men are poorly understood. With advancing age, testosterone levels gradually decrease in men, an effect associated with increasing fat mass, decrease in lean body mass, dyslipidemia, insulin resistance and adjustment in energy substrate metabolism. Aging is associated with a decline in metabolism, characterized by modifications in cardiac function, excitation-contraction coupling, and lower efficacy to generate energy. Testosterone deficiency -as found in elderly men- rapidly becomes an epidemic condition, associated with prominent cardiometabolic disorders. Therefore, it is highly probable that senior men showing low testosterone levels will display symptoms of androgen deficiency, presenting an unfavorable metabolic profile and increased cardiovascular risk. Moreover, recent reports establish that testosterone replacement improves cardiomyocyte bioenergetics, increases glucose metabolism and reduces insulin resistance in elderly men. Thus, testosterone-related metabolic signaling and gene expression may constitute relevant therapeutic target for preventing, or treating, age- and gender-related cardiometabolic diseases in men. Here, we will discuss the impact of current evidence showing how cardiac metabolism is regulated by androgen levels in aging men.

## Introduction

The multifactorial origin of cardiovascular diseases compels a comprehensive approach that incorporates lifestyle modification with an appropriate selection of medications for energy-regulation and its co-morbid conditions (1–4). In a physiological scenario, cardiometabolic adaptations involve a complex relationship among mechanisms responding to energy needs and substrate availability, in order to maintain homeostasis (5–7). During senescence, reduced ATP generation in the heart impairs normal contractile performance. There is a positive association between cardiac failure in age-related pathologies and insulin resistance, diabetes, sarcopenia and cardiovascular diseases (8, 9).

According to a 2019 update article from the American Heart Association, almost one in three adult men have some type of cardiovascular disease (10). Women are known to suffer cardiac disease 10–20 years later than men, which supports the hypothesis that physiological estrogen levels confer cardioprotective effects (11–14). In the past decades, the effect of sex-related steroid hormones on the cardiovascular system has been predominantly focused on estrogen actions, whereas research concerning the beneficial cardiac effects of androgens has been limited.

There is an extensive body of information indicating that administration of supraphysiologic doses of testosterone and cognate anabolic steroids induce adverse cardiovascular effects by triggering cardiac hypertrophy and heart failure (15). Although androgens have been considered previously to cause adverse cardiac outcomes, recent studies support favorable effects of these hormones on cardiovascular homeostasis (16–18). Many clinical publications over the past few years have indicated that very low levels of plasma testosterone are associated with pathophysiological processes, such as dyslipidemias, metabolic syndrome and diabetes type 2, which are considered as the underlying mechanisms involved in age-related cardiovascular diseases in men (19–23). Low circulating testosterone levels, as found in late-onset hypogonadism and elderly men, have also been associated with different types of heart diseases (24, 25). Moreover, epidemiological reports show that decreased testosterone concentration is a predictor of mortality in senior men (26).

A recent report from the Mayo Clinic (2018) exhaustively reviewed and analyzed the main clinical publications over the past 10 years related to testosterone levels, testosterone administration and their impact on the cardiovascular system (27). Pharmacological replacement of testosterone prevents heart disease, improves exercise-induced myocardial ischemia, dilates the coronary arteries, and decreases insulin resistance (28, 29). The overall evidence indicates that physiological testosterone levels are beneficial for the male cardiovascular system, while low testosterone concentration is linked to unfavorable metabolic profile and increased cardiovascular risk (27).

Aging, at same time, is associated with a gradual decline of testosterone levels in men (30). Plasma levels of androgens fluctuate throughout life. During childhood and before puberty, testosterone concentrations are usually lower in males than females. After puberty, testosterone levels increase in males, peaking at the age of 20–25. Thereafter, during aging, testosterone levels decrease (31–33). A cross-sectional study reported that in men over 40 years-old, total circulating testosterone levels decrease around 0.8% per year, while both free and albumin-bound testosterone levels decrease by 2%. In addition, plasma levels of sex hormone binding globulin (SHBG) increases by 1.6% per year, which may further decrease the bioavailable testosterone concentrations in elderly men (30, 34). Circulating SHBG levels in humans are influenced by different factors, such as nutritional state, metabolism, hormonal factors and aging (34–37).

Testosterone is well-known for both its androgenic properties and its anabolic effects. This steroid hormone induces changes on organs and tissues promoting the adoption of the adult male phenotype (38). In the heart, testosterone associates key physiological input for metabolism and protein synthesis (39). Cardio-specific and concentration-dependent effects of testosterone are modulated by its circulating plasma levels, cellular metabolism, modulation of intracellular transduction pathways and androgen receptor expression (15, 18).

Age-related andropause is characterized by diminished plasma testosterone concentration in adult men. With the increasing aging of the world population, andropause is quickly becoming an epidemic condition associated with metabolic disorders and prominent cardiovascular risks (32, 40, 41). Decreased testosterone concentrations in older men are linked to changes in body composition, like increase of fat mass and reduction of lean body mass, dyslipidemia, insulin resistance, and reduced glucose metabolism (22). The relationship between metabolic and cardiovascular risk in humans is evident in men suffering from hypogonadism, a condition in which the reduced functional activity of the gonads causes a decrease in testosterone levels (42). Hypogonadal men exhibit higher prevalence of cardiometabolic disorders compared to those with normal physiological levels of androgens (43). Retrospective studies of testosterone prescription databases have generated controversial and opposite results. Although testosterone replacement therapy to handle men hypogonadism it has been obtainable since 1939 (44), the apprehensions regarding the safety of testosterone treatment in men with cardiovascular diseases persist. However, to date, few systematic controlled studies have been performed to evaluate adverse events on cardiovascular system by testosterone administration (45–47). A recent randomized trial suggested that testosterone administration could increase cardiovascular risk in certain clinical populations, and it was suggested that pre-existing comorbidities as well as circulating lipid disturbances could influence the risk of cardiovascular events in older men. By contrast, several cross-sectional studies have demonstrated higher prevalence of cardiovascular diseases among men with low testosterone levels, and that replacement reduces cardiovascular risk (48, 49). Likewise, subjects with low plasma testosterone concentrations are more prone to develop insulin resistance and diabetes, as well as central obesity and heart failure (21, 50, 51).

Similar responses have been observed in elderly men exhibiting diminished testosterone concentrations, which result in hormonal and metabolic alterations associated with increased risk for developing cardiomyopathies (52). Androgen supplementation is a focus of emerging interest for the treatment of age-related metabolic diseases and muscle wasting (29, 53). Accordingly, male sex steroids can regulate cardiometabolic functionality and energy production through transcriptional and post-transcriptional mechanisms, and therefore, can offer insights into energy spreading pathways and their mechanistic control during aging (54). The mechanisms by which testosterone contributes to beneficial metabolic actions on the development of metabolic syndrome and diabetes type 2 are revised and discussed by Kelly and Jones and these effects seem to involve multiple targets of lipid and carbohydrate metabolism (55).

Additionally to testosterone reduction in elderly men, low concentrations of testosterone are also found in late-onset hypogonadism, reduction of testicular volume and malfunction of the androgen production machinery, systemic accelerated testosterone metabolism and expression of defective androgen receptors (24, 25, 42, 56). In skeletal muscle, physiological testosterone levels regulate a host of metabolic enzymes and transcription factors that regulate the expression of nuclear-encoded mitochondrial oxidative phosphorylation proteins (57). In the elderly, the ATP production machinery is less efficient, and this condition represents an energy dilemma. Metabolic unbalance during aging in men must be resolved by adjusting the energy substrates and the expression of metabolic genes (58–64). Age-related cardiac metabolic adaptations must regulate energy demands with fuel supply under switching nutrient conditions. The impact of testosterone administration to increase skeletal muscle mass is recognized, but its therapeutic use in aging men is still controversial and the underlying mechanisms remain to be defined. Recent reports indicate that testosterone therapy increases the expression of fibroblast growth factor 2 (FGF2) and decreases myogenic regulatory factor 4 (MRF4) and myostatin in skeletal muscle from men suffering hypogonadotropic hypogonadism, suggesting that the expression of these proteins contribute to muscle growth after testosterone therapy (65).

## Cellular Mechanisms of Testosterone Action

As it is well known, the hypothalamic-pituitary-gonadal axis modulates testosterone production. The hypothalamus produces and secretes gonadotrophin-releasing hormone (GnRH), which stimulates the pituitary to induce the pulsatile secretion of luteinizing hormone (LH), which then prompts the Leydig cells of the testes to produce testosterone (66, 67), which, in turn, exerts a negative feedback on GnRH and gonadotropin secretion. As age progresses, both the amount of Leydig cells and their ability to produce testosterone are reduced, contributing to lower circulating levels of androgens in elderly men (22, 30). However, other authors have argued that there is not a reduction of Leydig cell mass with aging, and that the main defect occurs in intracellular cell signaling and cholesterol transport (68). During obesity and aging, a raise in the activity of aromatase enzyme converts testosterone into estrogens in men, further reducing circulating plasma levels and the ability of testosterone to exert its appropriate metabolic actions (40, 69).

The main mechanism of action of testosterone involves direct binding to the intracellular androgen receptor (70–72), which is a member of the nuclear/steroid receptor superfamily. These receptors are proteins capable of binding their ligands in the cytoplasm or nucleus, and directly activating gene transcription (73, 74). The androgen receptor is a 110 kDa protein with three major functional regions for transactivation, a DNA binding domain and a hormone binding domain (75). After ligand binding, intracellular receptors are translocated to the nucleus, where they dimerize and bind to androgen response elements (ARE) to regulate target genes (74). Once bound to the hormone, other regulatory proteins or transcriptional coactivators can bind to the testosterone-androgen receptor complex to stabilize the promoter, thus achieving differential effects of this hormone either in a concentration-dependent or tissue-specific manner (76).

Previously, we and others have reported that testosterone also activates non-transcriptional signal transduction pathways, like extracellular signal-regulated kinase (ERK), phosphoinositide-3-kinase–protein kinase B/Akt (PI3K-PKB/Akt) and Ca^2+^-calmodulin-dependent protein kinase II (CaMKII) (77–80). In cardiomyocytes, testosterone induces hypertrophy through activation of the mechanistic target of rapamycin complex 1 (mTORC1) pathway (79) and glucose uptake by AMP-activated protein kinase (AMPK) activation (80). Overall, these evidences suggest that the effects of testosterone involve activation of anabolic and catabolic pathways. Thus, integration of transcriptional and non-transcriptional signals supplies cooperative mechanisms to support energy production under metabolic demand in cardiomyocytes.

As was mentioned above, SHBG is a protein that binds and transports testosterone within the bloodstream and regulates its bioavailability and access to extravascular target tissues (35, 81). Following the “free hormone hypothesis,” there is a proportion of testosterone bound to SHBG with high affinity, the rest corresponds to free testosterone which is either loosely bound to albumin, or unbound to proteins (82, 83). Free testosterone can cross the plasma membrane and it associates directly with androgen receptors; therefore, it is regarded as the bioavailable fraction, which is responsible for the biological activity of this hormone (84). SHBG levels have been negatively correlated with insulin levels (85), and in a meta-analysis that included cross-sectional and prospective observational studies, Brand et al. found an inverse relationship between total testosterone and free testosterone with SHBG levels, and metabolic syndrome (86, 87) raising the question about the role that intracellular androgen binding protein levels play in endocrine cellular physiology.

## Effects of Testosterone on the Cardiovascular System

Testosterone influences the cardiovascular system by acting directly on cardiac cells, the vascular tree, and by regulating cholesterol levels (88–90). In particular, exogenous administration of supra-physiological testosterone concentrations has been reported to produce cardiac hypertrophy, ventricular remodeling, cardiac failure, and sudden cardiac death (39, 91, 92). In humans and experimental animal models, testosterone has been related with higher risk of coronary artery disease through negative effects on plasma lipid and lipoprotein profiles, which may induce thrombosis and dilated cardiomyopathy (15, 39, 88–90, 93). It has been suggested that testosterone replacement therapy can increase blood viscosity and develop myocardial infarction, underscoring that in each individual patient with various comorbidities, one or more thrombosis mechanism/s may be playing an effect. However, a systematic review meta-analysis in men did not clearly show a significant association between testosterone use and higher risk of venous thromboembolism (94).

On the other hand, at normal physiological levels, androgen actions are necessary for a range of biological processes, including protein synthesis and cardiomyocyte metabolism. Androgens also induce other hemodynamic consequences, including vascular bed relaxation, thus reducing after-load and rapidly increasing cardiac contractility, which increases cardiac output (95). In humans, the effect of a 3-year testosterone administration did not increase atherosclerosis progression (96); however, another study showed that testosterone treatment of elderly men increased the volume of coronary artery plaques (97). The effects of androgen supplementation on plasma lipids depend on the dose, the route of administration and the subject population. In patients with congestive heart failure, testosterone would exert a beneficial role by improving functional capacity, cardiovascular parameters and quality of life (98). Interestingly, testosterone replacement therapy can reduce circulating levels of inflammatory mediators, including interleukin (IL)-1β and tumor necrosis factor α (TNF-α), as well as total cholesterol in patients with simultaneous coronary artery disease and testosterone deficiency (99, 100). The possible health risks and benefits of long-term testosterone replacement on older men with andropause caused by reduced testosterone concentrations are unknown. An interesting hypothesis has been postulated by Herring et el. suggesting that testosterone may simultaneously benefit and harm the cardiovascular system by different pathways (101). Caminiti et al. (102) reported that in elderly patients with congestive heart failure, testosterone replacement therapy improves functional capacity in, large-muscle strength, and glucose handling. The improvement of functional capacity and muscular strength are correlated with the higher plasma testosterone levels (102).

Hypertension is a risk factor for developing cardiovascular diseases. In adult men, hypertension is more frequent and occurs earlier than in women of similar age (103–105). In men, blood pressure rise has been associated with the effects and differences of sex-related steroid hormones. The different ranges in blood pressure in men, compared to women, remain until 60 years of age. Various epidemiological studies have reported that in men under 60 years old the systolic blood pressure is 6–7 mm Hg higher than in women, while diastolic pressure is higher by 3–5 mm Hg (106). On the other hand, in women over 60 years of age, blood pressure gradually increases, reaching a similar prevalence than in elderly men. The reduction of estrogens and the change in the estrogen/androgen ratio seems to be relevant for the increase in blood pressure in postmenopausal women (107, 108). An inverse relationship between systolic pressure and plasma testosterone levels has been reported in men, and an increased prevalence of hypertension in men with decreased free circulating androgens (104). The positive results of testosterone replacement are well documented. In randomized, double-blind, case-control clinical studies, the administration of hormones was associated with reduction of vascular tone (109). A beneficial role of testosterone was found in patients with congestive heart failure, by improving functional capacity, cardiovascular parameters, and quality of life (98). Several reports have suggested that testosterone vasodilatory action is mediated by the smooth muscle cell through ion channel modulation, modulating either potassium channel opening and/or calcium channel inactivation (110).

## Androgen Activates Intracellular Players Related to Cardiac Metabolism

The heart demands a continuous supply of energy to maintain muscle excitation-contraction coupling, and other intracellular adaptations, including fine-tuning in the expression of genes, ion homeostasis, signaling pathways, energetic balance and survival signals (58, 63, 111). Under normal conditions, cardiomyocytes prompt and effectively decode metabolic signals to evoke intracellular settings that improve cardiac functions to maintain an adequate energy balance that preserves work output and efficiency of the heart (112, 113). In the fetal period, glucose is the main energetic substrate for ATP generation in the heart, switching to fatty acid in adults to adjust to increased energy demands (63). Thus, in adult cardiomyocytes, under normal conditions, ATP is mostly produced by fatty acid β-oxidation. Glucose represents another substrate metabolized by glycolysis. Fatty acids are transported into the mitochondria by the enzyme carnitine palmitoyl transferase 1 (CPT-1). Glycolysis requires glucose uptake, which occurs in cardiac cells through glucose transporter 1 (GLUT1) and GLUT4 (113). Inside the cell, glucose can be phosphorylated by hexokinase and further metabolized to pyruvate. Both, β-oxidation and glycolysis produce acetyl-CoA to generate NADH and FADH2 via the citric acid cycle. These metabolites are later used by mitochondria to generate ATP through the electron transport chain. Aerobic respiration pathways by oxidative phosphorylation, produces up to 60% of their energy from fatty acid and triglyceride metabolism, 35% from carbohydrate metabolism, and 5% from amino acid metabolism. These metabolic pathways are regulated through substrate/product ratio, rate of enzyme action and gene expression of metabolic enzymes and transporters (9, 113).

Preference in energy substrate utilization may change in response to substrate availability or metabolic deregulation in cardiomyocytes (9, 113, 114). Under testosterone stimulation, the heart experiences a series of adaptive processes that enable acute metabolic changes for functional demands. If demand for increased effort is repeated or continuous, structural and metabolic changes occur (115). Dynamic adjustments of energy-generating machinery under either low- or high-testosterone inputs compel critical adaptive responses from cardiomyocytes to maintain work output and efficiency of the heart (63, 116, 117). Disturbed feedback between energy requirements and production impairs mitochondrial function and energetic efficiency of cardiomyocytes (118–120).

### Testosterone Improves Mitochondrial Function in Cardiac Cells

Transcriptional control of mitochondrial energy-generating machinery involves coordinated expression of proteins from two distinct genomes. Due to the limited coding capacity of mitochondrial DNA, nuclear encoded genes are also required (121). Mitochondrial enzymes are regulated through allosteric, post-translational, and transcriptional modifications (122). Testosterone regulates the expression of mitochondrial genes encoded by the nuclear genome and also, through direct action on mitochondria (123–125). Thus, by regulating cytosolic and mitochondrial pathways, testosterone exerts metabolic functions, with a possible feedback system between energy-producing mechanisms and cardiometabolic actions of testosterone in cardiomyocytes. Previous studies have shown that testosterone enhances the expression of mitochondria-encoded subunits of the respiratory chain, modulating mitochondrial respiratory function promoting functional efficiency (57, 126). In addition, androgens have direct interactions with respiratory chain complexes (123). In skeletal muscle cells, overexpression of androgen receptors increases mitochondrial enzyme activities and oxygen consumption (127). Following orchiectomy, young male mice show a decrease in the expression of genes associated with energy metabolism and oxidative phosphorylation, a phenotype that was reversed by testosterone treatment (61). With advancing age, androgen levels decrease and cardiac cells exhibit less mitochondrial number and lower energy production efficiency (57).

Androgen receptor signaling controls the transcription of several metabolic genes by engaging nuclear coactivator and corepressor proteins (128). In cardiac cells, peroxisome proliferator-activated receptor γ co-activator 1α (PGC-1α) stimulates mitochondrial biogenesis (129). PGC-1α is associated with cardiac energy metabolism through its upstream regulators and downstream targets (130) and it is highly expressed in the heart (131). The PGC-1α N-terminal domain interacts with proteins containing histone acetyltransferase activity, which allows remodeling of chromatin structure and transcriptional activation (132). Adjacent to the N-terminus, PGC-1α contains a regulatory domain with a LXXLL motif that interacts with nuclear receptors (133, 134). The PGC-1α C-terminus recruits proteins that facilitate its interaction with the transcription initiation machinery (135). Moreover, PGC-1α also regulates cardiac metabolism coactivating several transcription factor partners, including the androgen receptor (129). Also, testosterone up-regulates transcription of the nuclear respiratory factor-1 (NRF1), which controls the expression of mitochondrial respiratory chain complex proteins (136). NRF1 promoter contains putative ARE motifs in the DNA capable of binding the androgen receptor (125). It has been proposed that testosterone has a key modulatory role over NRFs and PGC-1α modulating mitochondrial biogenesis and metabolism (137). Moreover, androgens induce transcriptional and posttranslational regulation of Drp1, a key protein in the mitochondrial fission machinery (57, 123, 138). In contrast, low testosterone levels are associated with reduced expression of mitochondrial respiratory genes (126). In young male mice orchiectomy reduces the expression of genes associated with energy metabolism, oxidative phosphorylation, and ubiquinone pathways (139). Androgen receptor overexpression in cardiomyocytes increases mitochondrial enzyme activities and oxygen consumption (139). Testosterone administration, together with low-intensity physical exercise, increases mitochondrial biogenesis, increasing mitochondrial quality, and enhancing spontaneous physical activity, respiration and muscle mass (70). Therefore, the expression of metabolic genes related to testosterone may represent an important therapeutic modality to prevent or treat age- and gender-related cardiac diseases.

### AMPK and Cardiac Metabolism

AMPK is a serine/threonine kinase considered a fundamental intracellular energy sensor that regulates cell metabolism (6). AMPK is activated in response to physiological or pathological stimuli that reduce cell energy levels, by sensing the AMP/ATP ratio (140). AMPK modulates the activity of acetyl-coenzyme carboxylase, which in turn affects the levels of malonyl-coenzyme A, which is a key cellular energy regulator. AMPK coordinates metabolic pathways by limiting ATP expenditure and promoting ATP production to adjust to energy demands. In general, AMPK stimulates catabolic processes (141). Thus, AMPK promotes: (1) fatty acid β-oxidation, increasing their input to mitochondria and by activating enzymes such as carnitine palmitoyltransferase-1; (2) Glycolysis, increasing glucose uptake by GLUT4 and activating enzymes such as phosphofructokinase-2. Furthermore, activated AMPK can deliver energy status information through transcription factors to regulate gene expression of key proteins related to energy producing routes (142). A recent report has indicated that intramuscular injections of testosterone increase the expression and phosphorylation of AMPKα in adipose tissue and skeletal muscle biopsies of hypogonadism patients; these findings suggest that testosterone therapy may improve insulin sensitivity in obesity-associated hypogonadotropic hypogonadism men (143).

It has been well accepted that AMPK is cardioprotective (6). AMPK deficiency exacerbates cardiac necrosis and apoptosis following ischemic-reperfusion injury in transgenic mice expressing a dominant negative form of AMPK. Furthermore, the hearts of these mice show loss of contractile force and low ATP levels, suggesting that AMPK plays a crucial role in cardiac function (144, 145). Additionally, AMPK activation with AICAR blocks cardiac hypertrophy induced by several pro-hypertrophic stimuli, mainly by its inhibitory effect on the mTORC1 pathway (146). Metabolism during compensated cardiomyocyte growth implicates that anabolic processes are associated with controlled catabolic processes. In a prior work, we reported that stimulation of cardiomyocytes with testosterone during a short-time (<15 min) increases AMPK phosphorylation through CaMKII in a concentration- and time-dependent manner (80). Once AMPK is activated, GLUT4 translocation to the plasma membrane increases, thus increasing glucose uptake (147). Therefore, increased glucose uptake and utilization may be an adaptive response, because ATP production from glucose consumes less oxygen than that from fatty acids.

### Integrated Metabolic Actions of Testosterone and the AMPK/PGC-1α Axis in Cardiomyocytes

Metabolic information obtained through cytosolic energy sensors must be decoded by specific downstream metabolic pathways to improve energy production capacity. Moreover, PGC-1α interacts physically and functionally with well-known transcription factors involved in cardiomyocyte metabolism and growth (135). In fact, proximal PGC-1α promoter has different putative DNA binding sites to bind transcription factors involved in re-expression of gene programs during cardiac metabolism and cardiomyocyte growth, such as GATA4 and Myocyte-enhancer factor 2 (MEF2). Mutations in these transcription factors affect PGC-1α promoter activity (148, 149). Some authors have reported that MEF2C and histone deacetylase 5 (HDAC5) have both, positive and negative modulation of PGC-1α expression (135). Thus, PGC-1α represents a metabolic regulator by modulating gene expression and cell growth, suggesting that the activation of the AMPK-PGC-1α pathway is critical for the metabolic actions of androgens in the heart.

PGC-1α is activated by exposure to cold, fasting, exercise and various stimuli that promote oxidative metabolism (150, 151). Signaling pathways associated with these stimuli include p38 MAP kinase, β-adrenergic/cAMP, nitric oxide, AMPK, and CaMKII. These diverse pathways modulate PGC-1α activity by increasing PGC-1α expression, nuclear transactivation and its downstream regulated genes (141, 152, 153). In the heart, PGC-1α expression increases sharply at birth, coincident with a perinatal shift from glucose metabolism to fat oxidation (154). Different reports have indicated that low PGC-1α expression correlates with pathological energy mechanisms and heart failure (153, 155). In young or ovariectomized animals models, sex steroids control mitochondrial energy production modulating the transcriptional and post-transcriptional machinery (123).

In the heart of neonatal mice, overexpression of PGC-1α increases total mitochondrial mass (130, 156). In contrast, in adult mouse hearts, PGC-1α overexpression results in modest mitochondrial biogenesis, followed by cardiomyopathy associated with mitochondrial abnormalities (157). PGC-1α, together with PPARα, coactivates the enhancement of genes involved in the fatty acid β-oxidation pathway (116, 133, 158). Conversely, PGC-1α induces GLUT4 expression in skeletal muscle, resulting in increased glucose uptake, which, in turn, significantly reduces plasma glucose levels (159). Furthermore, normal mitochondria biogenesis is activated in response to changes in the ATP/ADP ratio and subsequent AMPK activation, which increases PGC-1α expression (156, 160). AMPK activation by AICAR increases β-oxidation of fatty acids by direct action on β-oxidation enzymes and by PGC-1α and PPARs activation. Additionally, in response to chronic energy deprivation, mitochondrial biogenesis is dependent on AMPK (6, 141). In prostate cancer cells, testosterone promotes cell growth in an AMPK-dependent pathway, which allows metabolic changes by increasing PGC-1α-dependent mitochondrial biogenesis (141, 161). In mice, treatment with testosterone increases PGC1α expression levels (136), while low levels of testosterone are associated with reduced expression of PGC-1α (125, 162). Furthermore, androgen receptor-deficient mice express low levels of PGC-1α (162).

### Effect of Sirtuins on Cardiac Metabolism

Protein acetylation/deacetylation play central roles in modulating cellular machinery related to metabolism (163). Mitochondria-mediated energy pathways contain acetylated proteins implicated in the tricarboxylic acid cycle, oxidative phosphorylation, fatty acid β-oxidation and glucose metabolism (163). In the heart, the protein sirtuin 3 (SIRT3) is a key regulator of mitochondrial function that adjusts energy availability, fuel sources and metabolic enzymes (164). Abnormal function of SIRT3 in pathophysiological processes is considered as the underlying mechanism of cardiovascular diseases (165–168). In cardiac cells SIRT3 is a stress-responsive deacetylase that protects these cells from damage induced by genotoxic and oxidative stress-mediated agents. It has been shown that the increased expression of SIRT3 protects murine cardiomyocytes from genotoxic and oxidative stress-mediated cell death (169, 170). Current evidence associates impaired SIRT3 activity with higher risk of aging-associated illnesses like cardiovascular disease (164, 171, 172). Therefore, altered expression of SIRT3 may be the consequence of impaired upstream metabolic signaling that influences PGC-1α activity, including AMPK and SIRT1 (129, 154). SIRT3 KO mice show cardiac mitochondrial function impairment and signs of premature aging (173). In addition, mice display contractile defects, such as a decrease of cardiac power, cardiac output, and developed pressure (171, 174). Porter et al. reported that decreased SIRT3 levels might raise the sensitivity of both heart cells and adult cardiac muscle to ischemia-reperfusion injury. This might contribute to a higher level of ischemia-reperfusion damage in the aged heart (175). Moreover, testosterone antagonizes doxorubicin-induced senescence of cardiomyocytes (176).

AMPK/PGC-1α interaction is critical for the up-regulation of mitochondrial function and SIRT3 activity (177, 178). SIRT3 can also deacetylate and activate liver kinase B1 (LKB1) that, on its own, increases the activity of AMPK. NAD+ is considered an inhibitor of cardiac hypertrophic signaling pathways and it is regulated to prevent cardiac hypertrophy and heart failure (6, 140). Interestingly, disruption of the CD38 gene in male mice enhances cardiac function by elevating serum testosterone levels and producing a general increase in NAD+ tissue concentration (179). A key metabolic regulator is AMPK, which controls mitochondrial homeostasis and metabolism by acting as an energy sensor (150, 159, 180). Moreover, the cytosolic deacetylate SIRT1 activates PGC-1α in cardiomyocytes to increase transcriptional activity and mitochondrial biogenesis (181). In the nucleus, androgen receptor signaling stimulates PGC-1α to increase the expression of various nuclear-encoded mitochondrial genes, including oxidative phosphorylation genes (137). SIRT3 is an important regulator of energy homoeostasis and basal production of ATP. The heart expresses high levels of SIRT3, leading to a marked reduction of ATP in its absence (182). However, SIRT3 can boost ATP levels in mitochondria due to the acetylation process, which diminishes with age (178). Aging-induced tissue fibrosis is mediated by Glycogen Synthase Kinase 3β (GSK3β) (183). Therefore, deacetylation of GSK3β by SIRT3 might reduce the tissue fibrosis associated to aging (165). Moreover, mitochondrial DNA content and activity, protein synthesis, oxidative capacity and ATP production are impaired by oxidative stress and free radicals. Regulated ROS production mediates redox signaling of transcription factors involved in mitochondrial biogenesis. However, an excess in the generation of mitochondrial ROS promotes oxidative stress that causes dysfunction and reduces mitochondrial biogenesis. Interestingly, SIRT3 reduces cardiac hypertrophy through increasing Foxo3a-dependent antioxidant defense mechanisms, suggesting that SIRT3 is an endogenous negative regulator of cardiac hypertrophy that protects the heart by suppressing cellular levels of ROS in mice (165). Thus, age-induced oxidative stress could be the underlying process that impairs mitochondrial biogenesis and downregulation of genes required for mitochondrial function and biogenesis induced by testosterone in cardiac cells.

A decline in cardiometabolic adaptations possibly reflects several age-associated changes, including a decrease in circulating testosterone levels (184). Thus, prevention of androgen deficiency might improve cardiovascular outcomes and extend longevity. Because cardiomyocytes must meet energy demands with fuel supply under switching nutrient conditions, the responses to androgen signaling in the elderly would not be able to produce enough ATP for anabolic effects, resulting in reduced energetic efficiency in cardiomyocytes. As was mentioned above, despite that testosterone controls gene-expression programs related to energy metabolism —a crucial requisite for the induction of energy-producing mechanisms in mitochondria— there is limited information about the signaling pathways interlinking metabolism and growth mediated by changes in circulating plasma testosterone levels and their effect on cardiometabolic homeostasis.

### Testosterone Metabolites in Aging

Testosterone can be transformed by the enzyme 5α reductase to 5α-dihydrotestosterone (DHT) mainly in skin, liver, hair follicles and prostate, where it acts locally (74). DHT is considered one of the main endogenous androgens (185). DHT binds to androgen receptors and induces the transcription of gene targets like testosterone. However, the dissociation constant of DHT-androgen receptor complex is 2–5 times lower than testosterone adduct, while DHT has a 10-fold higher potency on the signaling, which means that the effects of DHT and testosterone are different, but complementary (75).

Some reports suggest that DHT induces cardiac hypertrophy in cultured rat cardiomyocytes (186, 187) and in a rat model (188). On the other hand, treatment with finasteride, which inhibits the transformation of testosterone to DHT, reduces both cardiac hypertrophy and remodeling (187, 189). Evidence has indicated that the conversion of testosterone to DHT is required for mediating some of the effects of androgen on the cardiovascular system. In patients with mutations in type 2 5α reductase enzyme or finasteride treatment, the DHT levels are lower than healthy men but the androgenic phenotype is preserved. Nonetheless, these patients still show significant levels of circulating DHT. These results suggest that the conversion of testosterone to DHT is not essential for mediating its effects on muscle mass and strength (190). However, other studies have indicated that DHT may be an important risk predictor for cardiovascular disease in aging men. Healthy androgen levels are associated to survival and the total mortality of senior men displaying midrange concentrations of T and DHT is lower than men with low androgen levels, whereas those with higher DHT levels have shown lower ischemic heart disease mortality (191).

In men, estrogen levels increase during aging (192). Testosterone is converted to estradiol by the aromatase enzyme (193, 194), which is mainly expressed in adipose tissue (195). However, other factors also increase circulating estrogen levels, including impaired liver function, zinc deficiency, obesity, excessive use of alcohol and, environmental estrogens. Furthermore, estrogen levels are increased in men by various medications, such as statins and some blood pressure medications, antidepressants, and nonsteroidal anti-inflammatory drugs. In the case of obesity, aromatase activity increases estrogen levels and reduces testosterone levels (196). In turn, the generated estradiol exerts a negative feedback effect on LH secretion, further reducing plasma testosterone concentrations (192). In healthy men, pharmacological inhibition of aromatase reduces insulin sensitivity. Furthermore, patients with CYP19 aromatase mutations display reduced muscle and fat mass, and suffer insulin resistance (69, 196). Experimental gene selection data suggest that aromatization of testosterone to estradiol may be important in mediating the effects of androgens on body composition. The effects of testosterone on lean mass, muscle size, and strength were not reduced when its conversion to estradiol was inhibited by the treatment with aromatase inhibitors. However, the effects of testosterone on fat mass and sexual desire seemed to be mediated by estradiol (197). These results suggest that the different effects of sex hormones are complex and dependent on the relative levels of testosterone, DHT and estradiol, factors associated with health in elderly men. More studies are required to evaluate the mechanism by which androgens might influence the cardiovascular system in older men, in order to determine the risks and benefits of clinical intervention.

Given the important roles of androgens in normal physiology of men, abnormal low levels must be considered as one of the main causes implicated in several disorders and pathological conditions in aging men. In the context of human disease relevance, androgen deficiency treated with testosterone prescriptions at physiological concentrations has been associated with lower cardiometabolic risk and treatment outcomes. In 2015 the international expert consensus panel suggested that we need more research regarding the cardioprotective benefits of testosterone replacement, implying that there is enough evidence about the safety of testosterone therapy in hypogonadal and aging men and that the future research should be to study the suitable therapeutic options for age-related cardiovascular diseases (198).

## Conclusion and Future Research

Age-related cardiometabolic actions of testosterone are tightly regulated by its circulating plasma concentrations. This is an essential aspect regarding male physiology, since testosterone levels decline in older men, concomitantly increasing metabolic- and gender-related cardiovascular diseases. Further research on cardiometabolic testosterone effects are required to determine their effective cardiac properties. By applying controlled, randomized studies, working to attain physiological testosterone concentrations, we will obtain new data to understand the role of testosterone as a metabolic modulator that can improve ATP production, and, in parallel, increase cardiac performance. These further studies on the divergent energy-controlling mechanisms that mediate testosterone effects and testosterone-related metabolic gene expression, may represent an important therapeutic modality for preventing or treating gender-related cardiac diseases.

## Author Contributions

All authors listed have made a substantial, direct and intellectual contribution to the work, and approved it for publication.

## Conflict of Interest

The authors declare that the research was conducted in the absence of any commercial or financial relationships that could be construed as a potential conflict of interest.
